# Anti-cancer peptides: classification, mechanism of action, reconstruction and modification

**DOI:** 10.1098/rsob.200004

**Published:** 2020-07-22

**Authors:** Mingfeng Xie, Dijia Liu, Yufeng Yang

**Affiliations:** 1 Department of Bioengineering, Zunyi Medical University Zhuhai Campus, Zhuhai, Guangdong 519040, People's Republic of China; 2 Zhuhai Key Laboratory of Fundamental and Applied Research in Traditional Chinese Medicine, Zunyi Medical University Zhuhai Campus, Zhuhai, Guangdong 519040, People's Republic of China

**Keywords:** anti-cancer peptides, classification, mechanism of action, reconstruction, modification

## Abstract

Anti-cancer peptides (ACPs) are a series of short peptides composed of 10–60 amino acids that can inhibit tumour cell proliferation or migration, or suppress the formation of tumour blood vessels, and are less likely to cause drug resistance. The aforementioned merits make ACPs the most promising anti-cancer candidate. However, ACPs may be degraded by proteases, or result in cytotoxicity in many cases. To overcome these drawbacks, a plethora of research has focused on reconstruction or modification of ACPs to improve their anti-cancer activity, while reducing their cytotoxicity. The modification of ACPs mainly includes main chain reconstruction and side chain modification. After summarizing the classification and mechanism of action of ACPs, this paper focuses on recent development and progress about their reconstruction and modification. The information collected here may provide some ideas for further research on ACPs, in particular their modification.

## 1. Introduction

Cancer comprises a collection of diseases caused by excessive proliferation of cells in the body that cannot be effectively regulated [1]. So far, most cancers, in addition to chronic leukaemia, cannot be permanently cured. According to statistics, deaths from cancer reached 9.56 million cases globally in 2018, and there were 18.07 million new cancer cases that year. In China, the aforementioned cases were 2.86 million and 4.28 million, respectively [2]. These data suggest that current treatments for cancer are still inadequate.

At present, the main therapeutic strategies for cancer include surgery, radiotherapy, chemotherapy and immunotherapy. Surgical treatment can, in general, quickly remove obvious solid tumours; this may be an effective therapeutic method for early or even middle tumours. However, this kind of treatment often leads to obvious trauma, bleeding, infection, weakened immunity and other risks, and is therefore not the best choice for most patients with advanced tumours [3]. Radiation therapy is often used in patients with cancer who have not benefitted from surgical treatment. However, radiotherapy often results in a series of complications, and this treatment method is expensive and the course of treatment is long [4]. Chemotherapy is a systemic therapy that involves the introduction of chemicals into the body to attack cancer cells. Long-term use of this therapy increases proneness to drug resistance with very high possibilities of recurrence; moreover, drugs kill tumour cells and normal cells indiscriminately, resulting in obvious side effects [5]. Immunotherapy modulates the patient's own immune system to facilitate an anti-tumour effect. The effect of this treatment is long lasting, and the side effects are less than with chemotherapy [6]. In recent years, many types of immunotherapy, including PD-1/PD-L1 immunotherapy and chimeric antigen receptor gene-modified T (CAR-T) cell immunotherapy, have been introduced in clinical trials or have been marketed [7,8]. PD-1/PD-L1 immunotherapy, which kills cancer cells by activating their own immune system, is promising. However, the curative effect varies between individuals. Even worse, it may sometimes lead to autoimmune myocarditis [9]. CAR-T cell immunotherapy is a therapeutic strategy, which obtains T cells that are then modified with chimeric antigen receptor gene so as to express tumour cell-specific antigen receptors. When transfused into patients after *in vitro* amplification, these so-called CAR-T cells can recognize and kill tumour cells [10]. This treatment strategy has demonstrated efficacy in haematological malignancies and other cancers. However, because CAR-T cells have immunological memory, they can exist in the body for an extended period and may be overactivated. In addition, the potential of triggering cytokine storm remains a serious challenge that needs to be addressed in CAR-T cell therapy [11]. In short, current clinical treatment methods have their own advantages, disadvantages and scopes of application. It is difficult for a single treatment method to achieve satisfactory curative effects, and combined therapy can, to a certain extent, offer better overall cure. Therefore, research and development of novel therapeutic methods or anti-tumour drugs is an urgent unmet need.

With the advent of molecular biology, a large number of short peptides have been found to exist in a wide range of organisms [12]. These peptides can kill bacteria, fungi and tumour cells, and even regulate the immune system. The accumulation of structural and functional data has seen the emergence of cationic low-molecular-weight peptides with anti-tumour activity, which are classified as anti-cancer peptides (ACPs) [13]. In fact, cationic peptides isolated from various organisms were historically assessed for antimicrobial activities and were studied as such prior to their first being described as potent anti-cancer agents in 1985 [14]. Due to their unique mechanism of action, ACPs have many advantages over conventional chemotherapy, which can better inhibit tumour cell proliferation, migration and tumour angiogenesis [15]. With mature solid-phase synthesis technology, ACPs have a low cost of production, and are easy to modify. Coupled with the merits of relatively high tissue penetration and low occurrence of drug resistance, the clinical application prospect of ACPs are promising. A search of the US National Institutes of Health Clinical Trials database (https://clinicaltrials.gov/) using the phrase ‘anti-cancer peptides’ found 1002 peptide-based clinical trials that targeted different types of cancer [16]. For example, Bryostatin 1, one of the most abundant and best-studied peptides of the bryostatin family, has shown anti-tumour activity in Phase I trials in patients with malignant melanoma, lymphoma and ovarian carcinoma [17,18]. Aplidine (plitidepsin) is well tolerated in clinical trials, with low toxicity in completed Phase I clinical trials, and Phase II studies are currently occurring. In a Phase II clinical trial, aplidine was tested against advanced medullary thyroid carcinoma, advanced malignant melanoma, small cell lung cancer and advanced renal cell carcinoma [19]. As of November 2019, more than 20 ACPs have been approved by FDA and EMA. ACPs like Kyprolis, SomaKit TOC, Lutathera and Gallium Dotatoc Ga68 have been marketed in recent years [16]. Collectively, ACPs represent a promising alternative to conventional chemotherapy. However, many ACPs also have some disadvantages, including substantial toxicity and poor targeting, which seriously impair their potency. Therefore, mechanisms of effective reconstruction or modification of ACPs so as to improve their therapeutic properties and reduce their toxicity have become a major research focus. In this paper, the classification, mechanism of action and methods of modification of ACPs are reviewed. This review aims to provide useful references for the modification of ACPs and the development of peptide anti-tumour drugs.

## 2. Classification of ACPs

**Figure 1.**
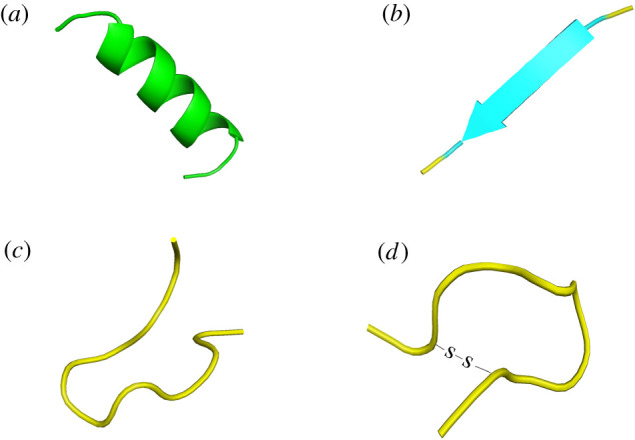
Schematic diagram of ACPs' structural classification: (*a*) α-helical; (*b*) β-pleated sheets; (*c*) random coil; (*d*) cyclic ACPs.

Since the discovery of ‘cecropins’ by Swedish scientist Boman and colleagues in 1980 [20], abundant bioactive peptides have been continuously found, some of which have various biological functions such as anti-tumour effects and regulation of the immune system [12]. There are different species of ACPs that have been identified from various organisms [21], which are classified in several ways. Since the activity of ACPs depends largely on the type, number and structure of their amino acids, structural classification is the most common method of classification at present [22]. According to this classification, ACPs can be divided into four categories: α-helical, β-pleated sheets, random coil and cyclic [23] (figure 1).

### 2.1. α-helical ACPs

In α-helical ACPs, the peptide chain is generally short in length and simple in structure. It is the most typical structure type of ACPs and exists widely in the epidermis of amphibians [24]. Magainin II from the African clawed frog was the first α-helical ACP found to have anti-cancer activity. Its half maximal inhibitory concentration (IC_50_) in lung cancer cell A549 is 110 µg ml^−1^; it is not toxic to human immortalized epidermal cells under this condition [25,26]. Another α-helical peptide, named Aurein, was obtained from glandular secretions of green and golden bell frogs and southern bell frogs [27]. Aurein 1.2 has shown substantial anti-cancer activity and has demonstrated strong inhibitory effects on T98G glioblastoma cells, at an IC_50_ value as low as 2 µM [28]. In recent years, an increasing number of α-helical ACPs have been discovered, but not all have robust anti-cancer effects. For example, L-K6 exerts an inhibitory effect on human breast cancer MCF-7 cells, with IC_50_ values up to 30.2 µM [29]; the IC_50_ values of LL37 and FK-16 on human colorectal cancer cells HCT116 are up to 40 µM and 30 µM, respectively [30]. The α-helical ACPs are the most extensively studied type of ACPs at present. Although most of them have adequate inhibitory effects on tumour cells, ACPs also have several drawbacks, such as cytotoxicity and side effects. Our group has designed and synthesized a series of α-helical ACPs that possess excellent inhibitory effects on a variety of tumour cells, with IC_50_ values reaching the order of magnitude of micromoles; nevertheless, these peptides also have relatively high toxicity against some normal cells. Hence, the reconstruction of these ACPs is warranted, for which further in-depth research is necessary.

### 2.2. β-pleated sheet ACPs

Most of the β-pleated sheet ACPs have two or more disulfide bonds, with good stability. These structures are more complex than α-helical ACPs and are found mainly in plants and animals [31]. Bovine lactoferrin (LfcinB), which comprises an important part of the bovine immune system, is a typical β-pleated sheet ACP [32]. Its IC_50_ value in gastric cancer cell line MGC803 was found to be 32 µM [33]. MPLfcinB6, which was formed by connecting seven arginines to LfcinB through glycine–glycine ligands, could kill human T-leukaemia cells quite effectively and has an IC_50_ value of 25 µM, half of that before modification [34]. A researcher obtained another peptide, modified from LfcinB, called LfcinB-P13, which could better promote apoptosis of the hepatocellular carcinoma cell line HepG2. Its IC_50_ value was 50 µg ml^−1^, which is better than that of LfcinB (IC_50_: 70 µg ml^−1^) [35]. Human neutrophil peptide (HNP-1) is also a typical endogenous β-pleated peptide, which has a strong inhibitory effect on the human prostate cancer cell line PC-3, the IC_50_ value of which is as low as 2.2 µM [36]. On the whole, the anti-tumour activity of β-pleated ACPs is generally lower than that of α-helical ones, but β-pleated ACPs are less toxic to normal tissue cells; hence, they have good future prospects for development.

### 2.3. Random coil ACPs

Random coil ACPs are generally rich in proline and glycine, and lack a typical secondary structure [37]. Alloferon, a type of glycine-rich random coil ACP derived from insects, can stimulate the activation of NK cells and interferon synthesis in animal and human models, which further enhance antiviral and anti-tumour abilities in mice and humans [38]. It has been proven to have therapeutic value. In patients infected with herpes simplex virus and human papillomavirus, the antiviral and immunomodulatory effects of Alloferon have been clinically proven [39]. Based on LFcinB_18–28_, a new peptide, KW-WK, was designed by introducing arginine and tryptophan, which revealed an irregular coil in a simulated aqueous environment, and did little damage to human kidney 293 cells even when the concentration was 128 µM; toxicity of the template peptide began to appear at 64 µM [40]. PR-39, which is rich in proline arginine, is an irregular curly antimicrobial peptide derived from neutrophils. It has moderate inhibitory effect on tumour cells, but has a strong inhibitory effect on normal human embryonic kidney 293T cells [37,41]. In order to reduce its cytotoxicity, a mutant based on PR-39, named PR-35, was designed, whose cytotoxicity was evidently decreased, but the biological activity of which was the same as the template peptide. The IC_50_ value of PR-39 in 293T cells was 16 µg ml^−1^; for PR-35, however, 90% of 293T cells still survived at this concentration [41]. Although the killing effect of random coil ACPs on normal cells was much lower than that of other types of ACPs, their inhibitory effect on tumour cells was worse than that of the first two types. Therefore, improving their anti-tumour activity is the focus of this research.

### 2.4. Cyclic ACPs

Cyclic ACPs are closed peptides composed of a head-to-tail cyclization backbone or disulfide bonds that form cystine knots; they are more stable than linear structures [42]. Three new cyclic peptides, Diffusa Cytide 1–3, were found in the leaves and roots of the white snake plant, and have particularly strong inhibitory effect on three kinds of prostate cancer cells; furthermore, they can inhibit the migration of prostate cancer cells *in vitro* at a concentration of 0.05 µM [43]. Currently, cyclic ACPs account for the majority of ACPs in clinical studies; these peptides have a strong inhibitory effect on cancer cells [44]. H-10, a novel cyclic pentapeptide, has a concentration-dependent inhibitory effect on mouse malignant melanoma B16 cells, with an IC_50_ value of 39.68 µM with no toxicity to human peripheral lymphocytes and rat aortic smooth muscle cells [45]. RA-XII, a natural cyclic peptide, derived from *Taxus yunnanensis*, could inhibit colorectal tumour growth and metastasis through the AMPK/mTOR/P70S6 K and PI3 K/AKT/NF-kB pathways; its IC_50_ value is 5 µM [46]. In conclusion, cyclic ACPs are types of ACP with better anti-cancer activity and lower toxicity than other ACPs; they may be a useful reference for the effective modification of ACPs.

## 3. Anti-tumour mechanism of ACPs

### 3.1. ACPs destroy the structure of cell membrane

**Figure 2.**
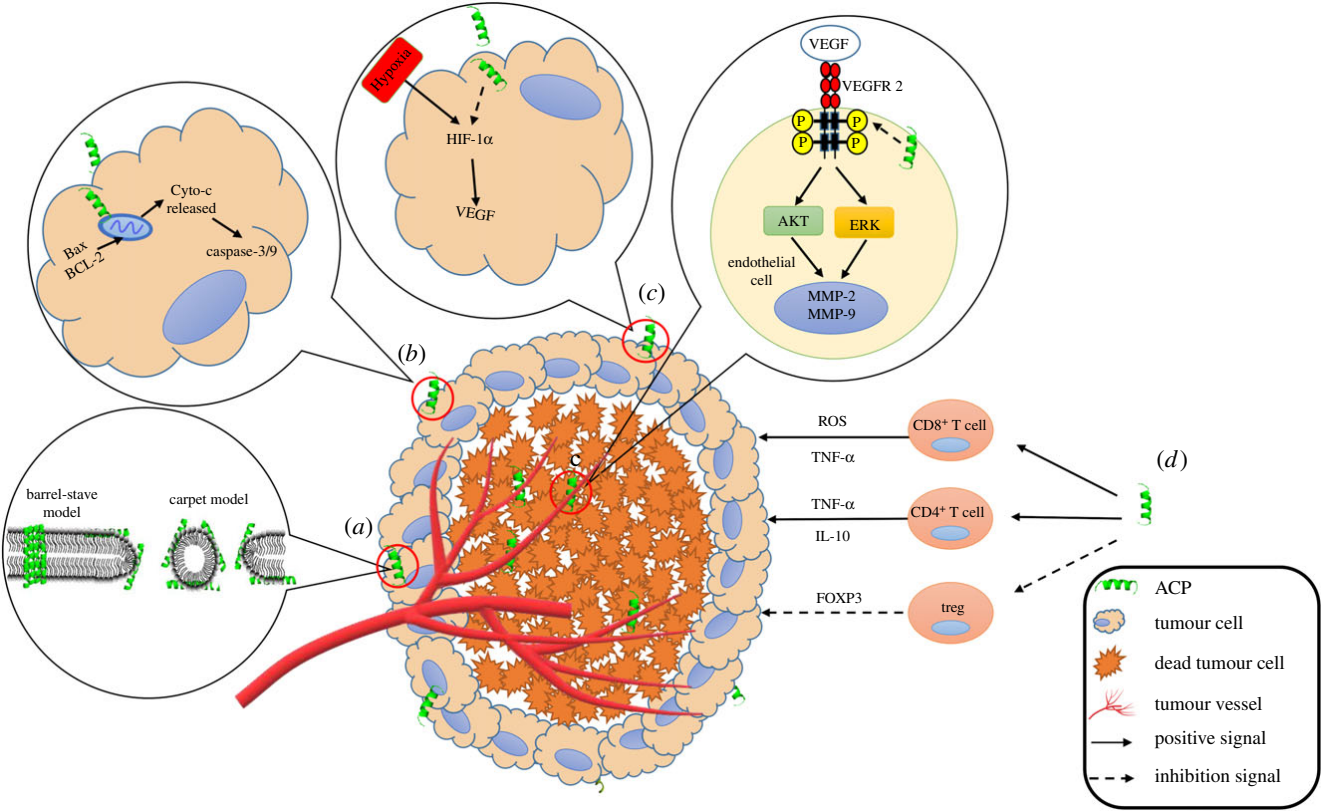
Schematic diagram of anti-tumour mechanism of ACPs: (*a*) destroy the structure of cell membrane; (*b*) apoptosis; (*c*) inhibiting angiogenesis; (*d*) immune regulation.

As early as 1977, Ehrenstein & Lecar proposed the ‘barrel-stave’ model, believing that the main mechanism of action of ACPs is to cause cell membrane fragmentation or apoptosis by depolarization of the cell membrane, leading to failure of tumour cells to maintain normal osmotic pressure [47]. Subsequently, Pouny *et al.* proposed a ‘carpet’ model, which suggested that ACPs caused cell death by destroying the cell membrane of cancer cells, leading to massive leakage of cytoplasmic contents [48]. Most ACPs act directly through this mechanism (figure 2*a*), which endows them with unique advantages over conventional chemotherapy. Unlike conventional chemotherapy, many ACPs can kill both the metabolically active tumour cells and slow-growing ones, as well as multidrug-resistant ones [15]. HPRP-A1-TAT, a hybrid peptide, can destroy the cell membrane to cause rapid leakage of cytoplasmic contents, and has a strong anti-cancer activity. The IC_50_ value of this ACP in melanoma, gastric cancer, liver cancer and cervical cancer cells is less than 10 µM [49]. Temporin-La is an ACP derived from bullfrog skin, which enters into cells and exerts its anti-cancer activity by destroying the tumour cell membrane. Its IC_50_ value in liver cancer cells is 11.19 µM, and it has no obvious toxicity to normal liver tissue cells [50]. Progressive accrual of cases indicates that the mechanism of anti-tumour activity of ACPs is universal; however, the mechanism of detailed interaction between ACPs and the cell membrane needs further study.

### 3.2. Apoptosis

With more in-depth studies, researchers have found that ACPs can also lead to the release of cytochrome C (Cyto-c) and induce apoptosis by destroying the mitochondrial membrane of tumour cells. For example, the ACP Ra-V triggers mitochondrial apoptosis by mediating the loss of mitochondrial membrane potential, the release of Cyto-c and the activation of the caspase apoptotic pathway, leading to apoptosis of human breast cancer cells [34] (figure 2*b*). Dolastatin 10 isolated from the marine mollusk *Dolabella auricularia*, and it exhibited remarkable *in vitro* cytotoxicity against a number of human cancer cell lines, such as melanoma, sarcoma, colorectal cancer and ovarian cancer cells. Further studies showed that Dolastatin 10 can induce apoptosis in many tumour cell lines in which the pro-apoptotic molecule Bax is upregulated and the anti-apoptotic molecule Bcl-2 is downregulated [51]. The *Bacillus subtilis* lipopeptide could inhibit the growth of K562 myelogenous leukaemia cells and induction of apoptosis by causing ROS burst, and induction of the intrinsic pathway indicated by the upregulated expression of Cyto-c, Bax and Bad, together with downregulated expression of Bcl-2 [52]. Collectively, inducing mitochondrial-dependent apoptosis is one of the important ways for ACPs to exert an anti-tumour effect.

### 3.3. Inhibition of tumour angiogenesis

Tumour cells can induce vascular endothelial cells to form new blood vessels through high expression of vascular endothelial growth factor (VEGF), which promotes tumour growth and metastasis. Tumour cells without neovascularization have a slow growth rate [53]. KV11, a peptide with 11 amino acids, present in the apolipoprotein (A) KV domain of the anti-angiogenesis functional domain, reduced angiogenesis by inhibiting migration of human umbilical vein epithelial cells (HUVECs) and formation of microtubules. The IC_50_ value is 15 µM and this ACP has no lethal effect on HUVECs. In a severe combined immune deficiency mouse transplanted tumour model, 100 µM of KV11 peptide did not have a significant effect on the growth and proliferation of breast cancer tumour cells, but inhibited tumour growth by inhibiting tumour angiogenesis [54]. Two cyclic peptides, PF1171A (1) and PF1171C (2), isolated from the soil fungus *Penicillium* sp. FN070315, significantly restrained VEGF-induced migration, invasion, proliferation and tube formation of HUVECs, as well as neovascularization. The experimental results showed that these two cyclic peptides play an antiangiogenic role by downregulating both the expression of hypoxia-inducible factor-1α and the phosphorylation of VEGF receptor 2 [55] (figure 2*c*). Another ACP, Temporin-1CEa, was found to inhibit the formation of human VEGF in human melanoma A375 cells, thus inhibiting the formation of new blood vessels at an IC_50_ value of 18.2 µM [56]. Briefly, through this mechanism of action, ACPs do not directly kill tumour cells, but instead inhibit neovascularization; hence, they have minimal side effects on normal cells. Therefore, ACPs of this kind have good prospects for clinical application.

### 3.4. Immune regulation

Bovine lactoferrin (LfcinB) is a cationic peptide derived from lactoferrin, which can induce the production of cytokines so as to enhance host defence against the tumour. This is a way to restrain the growth of cancer through immune regulation [57]. Immunohistochemical analysis of the tumour showed that lymphocytes in treated animals increased to a magnitude of 20 times compared with untreated animals; that is, the ability of lymphocytes to infiltrate the tumour increased significantly. When CD3^+^ cells were exhausted in mice, all LfcinB-induced tumour inhibition was abrogated. Therefore, LfcinB inhibition of head and neck squamous cell carcinoma growth was achieved through immune regulation [58]. MENK is an endogenous neuropeptide that plays a role in tumour immune response by upregulation of CD8^+^ T cells' activity and induction of dendritic cell maturation to initiate T cell response, and intensifies CD4^+^ T cell functions as well as the secretion of various cytokines. Among others, the expression of forkhead box P3 transcription factor (FOXP3) is inhibited, which reduces the regulatory T cell (Treg) levels *in vivo* and greatly enhances the anti-tumour effect [59] (figure 2*d*). Other studies have shown that MENK also plays an important role in the neuroendocrine and immune system. It exerts anti-tumour activity by binding to opioid receptors on immune cells and cancer cells, and acts as an immune booster [60]. In addition, MENK can inhibit the proliferation of human cancer cells through cyclin-dependent kinase inhibition pathways [61]. ACPs can enhance the immune system of the body through an immunomodulatory mechanism and inhibit the growth of tumours; this could be an avenue for further research.

## 4. Reconstruction and modification of ACP

As potential candidates for cancer therapy, ACPs have many obvious advantages, but also have several shortcomings, which may severely hamper and slow down their use in clinical research and application. In recent years, several researchers have devoted themselves to the reconstruction and modification of ACPs, hoping to retain their advantages while reducing their related side effects, thereby improving their therapeutic properties. The reconstruction of ACPs is mainly divided into main chain reconstruction and side chain modification. Main chain reconstruction primarily refers to the replacement of natural or non-natural amino acids, whereas side chain modification primarily includes cholesterol modification, phosphorylation, polyethylene glycol (PEG) modification, glycosylation and palmitoylation [62–64] (table 1).

**Table 1.** Type of reconstruction or modification in ACPs.

peptide	type of reconstruction or modification	effect (IC_50_: before/after)	references
S25 K	amino acid substitution	cervical cancer cell: 13.2 µM/1.4 µM	[65]
A6c-Gly-Tic/Oic	non-natural amino terminal substitution	lung and breast cancer cell: 112.5 µM/7.5 µM	[66]
HAL-C	cholesterol modification	ovarian cancer cell: 54.1 µM/12.7 µM	[67]
Tat-NYYRK	phosphorylation modification	breast cancer cell: 1 mM/500 µM	[68]
Temporin-1CEa-LIP	PEG modification	breast cancer cell: 29.32 µM/29.32 µM	[69]
R-lycosin-I	glycosylation	lung cancer cell: 37.4 µM/9.6 µM	[70]
C16-P_Cat_P_Hex_P_Hex_P_Cat_-NH2	palmitoylation modification	colorectal cancer cell: 0/12.5 µM	[71]

### 4.1. Main chain transformation

#### 4.1.1. Replacement of natural amino acids

The type and sequence of amino acids have a great influence on the structure and function of short peptides. Therefore, changing the type of main chain amino acids is the most common and effective way to modify ACPs. Amino acid substitution often causes changes in net charge, hydrophobicity and helicity, leading to changes in activity and selectivity of ACPs [72,73]. Tumour cells tend to be more electronegative than normal cells, whereas ACPs are generally positively charged and can interact with them well. In addition, the hydrophobic interaction between the hydrophobic surface of ACPs and extracellular phospholipids of tumours can be quite strong; reasonable balance of these multiple effects could remarkably enhance the anti-cancer activity of ACPs [74]. To investigate the effect of net charge on the activity of ACPs, K7S peptide was modified by mutual substitution of serine and lysine on the hydrophilic surface to obtain a series of mutants, in which the net charge changed from +4 to +10, while their hydrophobicity remained unchanged [65]. The experimental result showed that the IC_50_ value of mutants in cervical cancer cells decreased significantly to 1.4 µM from 13.2 µM, thereby reducing toxicity to normal cells. With reference to hydrophobicity, a series of modified peptides were obtained by replacing the alanine of peptide V13KL with a more hydrophobic leucine to enhance its hydrophobicity, or by replacing the leucine with an alanine to reduce its hydrophobicity. However, excessive hydrophobicity (A12 L/A23 L/A20 L) increases the toxicity of normal cells, whereas hypo-hydrophobic property (L6A/L21A/V13 K) leads to decreased selectivity [75]. Besides hydrophobicity, the helicity of ACPs can also be clearly changed by amino acid substitution [76]. For example, a new peptide, MEL-pep, was synthesized by replacing the 8th valine and 14th proline with lysine in Melitin (MEL), which greatly enhanced its charge and helicity. This change decreased the IC_50_ value of MEL-pep in hepatoma cells to 4.44 µM, less than half of that of the original MEL (IC_50_: 11.09 µM), and its toxic and side effects were significantly reduced [77]. Changing of net charge, hydrophobicity and helicity of ACPs are now commonly implemented to optimize anti-tumour activity of ACPs. However, the exact structure–function relationship remains elusive, which is the reason for the low efficiency of current research on the modification of ACPs and could perhaps be the focus of future research.

#### 4.1.2. Substitution of non-natural amino acids

Non-natural amino acids often have physico-chemical properties that are not found in natural amino acids. Therefore, these properties can be used to develop short peptides with special properties that can better interact with cell membranes [78]. The substitution of non-natural amino acids with special physiochemical properties in the main chain can effectively improve the selectivity of ACPs and reduce their cytotoxicity, thus improving the therapeutic index [79]. Compared with natural amino acids, non-natural amino acids have three main advantages: (i) they have a variety of physiochemical properties [80]; (ii) non-natural amino acids provide greater control of the conformational flexibility of the peptides, thus increasing the potential for organism selectivity and potency [81]; and (iii) they have higher metabolic stability [79]. Researchers have synthesized a new set of short peptides with the use of tetrahydroisoquinoline carboxylic acid (Tic) instead of phenylalanine, octahydroindolecarboxylic acid (Oic) instead of proline, 1-aminocyclohexane carboxylic acid (A6c)/A5c instead of alanine or leunine and 2,4-diaminobutanoic acid/2,4-diaminopropionic acid instead of lysine to transform the template peptide; the experimental results showed that the modified A6c-Gly-Tic/Oic had 15 times higher anti-tumour activity, the IC_50_ value in 12 types of cancer cells was less than 7.5 µM, and it was non-toxic to normal cells [66]. Another non-natural amino acid used in ACPs modification is d-amino acid. Studies have shown that the use of d-amino acids to replace l-amino acids resulted in changes in the structure of the polypeptide, which reduced the haemolytic rate of normal cells and significantly improved stability and inhibitory activities [82]. Four peptides, PMI-1/4, were obtained by a single replacement l-serine with four kinds of d-amino acids in the carboxyl terminal of PMI. These peptides have improved stability and inhibitory activities, among which PMI-4 showed the strongest inhibitory activity against U87 cells, with IC_50_ value (30.9 µM) reduced by half compared with the original peptide [83]. In addition, a series of unique analogues of Bactenecin peptides were synthesized that used lysine instead of arginine and tryptophan as an alternative to valine, and then replaced all the d-amino acids with l-amino acids. Compared with the original peptide, the peptide analogues had greatly reduced haemolytic activity when the concentration was up to 100 mM. The series of analogues of erythrocytic haemolytic activity are less than 3%, and their stability is improved significantly [84]. In conclusion, the introduction of non-natural amino acids has greatly enriched the modification methods of ACPs and obtained many satisfactory new peptides or analogues, which have effectively promoted the modification and clinical application of ACPs.

### 4.2. Side chain modification

#### 4.2.1. Cholesterol modification

Cholesterol is a component of the animal cell membrane. The incorporation of cholesterol into ACPs possibly drives the self-assembly of peptides, which may facilitate the entry of ACPs into the cancer cell [85]. HAL-B/C/D are a series of short peptides obtained by cholesterol modification of the short peptide HAL-2, among which HAL-C has the best modification effect. The IC_50_ value in ovarian cancer SKVO3 cells was reduced from 54.1 to 12.7 µM [67]. Peptides B1-I/II/III and IV, another series of short peptides, were designed by introducing cholesterol at the N-terminal of B1, thereby improving anti-cancer activity. Among these, B1-I effectively inhibits human breast cancer MCF-7 cells, human erythroleukemia K562 cells and human prostate cancer DU145 cells at IC_50_ values of 3.1 µM, 3.6 µM and 4.3 µM, respectively. The anti-cancer effect of B1-I is greatly enhanced. At the same time, it is less toxic to normal cells; the IC_50_ values in GES-1 and HEK-293 cells are 51.6 µM and 48.8 µM, respectively. Modification with cholesterol greatly improves the selectivity of B1-I for tumour cells and obviates drug resistance in some circumstances [86]. In brief, cholesterol modification may be an effective way to improve anti-tumour activity and reduce cytotoxicity of ACPs.

#### 4.2.2. Phosphorylation modification

Phosphorylation is a pervasive modification of the protein or peptide after being synthesized, which occurs at several specific phosphorylation sites in amino acids, such as threonine, tyrosine, serine and so on, in general [87,88]. Studies have shown that the peptides Ac-NIYQT-NH_2_ and Ac-NYYRK-NH_2_ were obtained by phosphorylation of aspartic acid/aspartic amide and glutamic/glutamine of parent peptides, Ac-DIYET-NH_2_ and Ac-DYYRK-NH_2_, at phosphorylation sites. The two peptides bound to the human immunodeficiency virus type I reverse transcription activation protein (Tat) [68]. Among them, the IC_50_ value of Tat-NYYRK in breast cancer cells is 500 µM; for the rest of the peptides, IC_50_ values are greater than 1 mM, but all the peptides are non-toxic to normal cells at high concentrations. p-Peptide1/2/3, three new peptides, were obtained by binding the template peptide with cell penetrating peptide after serine phosphorylation at the serine site [89]. The IC_50_ value of these three peptides in human glioma cells U251 and H4 was 50 µM, and compared with the original peptide (IC_50_: 150 µM), the anti-cancer effect was greatly improved. So far, there are few studies on direct phosphorylation modification of amino acid sites in ACPs. However, existing data show that this modification is not as effective as the other modifications in enhancing anti-tumour activity, but it can significantly reduce the toxic and side effects of ACPs. Therefore, it can be used as an alternative type of ACP modification.

#### 4.2.3. Polyethylene glycol modification

PEG modification is the coupling of PEG groups with free side chain groups on the molecular surface of ACPs through covalent bonds to change the physico-chemical properties of ACPs, thereby improving their selectivity and reducing their toxicity [90]. As PEG is a macromolecule, it can often increase the molecular diameter of ACPs, thus extending their half-lives [91]. Temporin-1CEa-LIP, a new drug carrier system, was established by PEG modification of Temporin-1CEa template peptide, and the modified peptide exhibited an unchanged IC_50_ value in breast cancer cells (29.32 µM), but its serum stability was greatly improved [69]. On the other hand, PEG modification may endow ACPs with superior targeting ability. Compared with the template peptide, PEG-CREKA achieved better targeting of cancer cells, and the accumulation amount in HeLa cells of cervical cancer was far more than the template peptide, and it was not toxic to normal cells [92]. PEG modification has some issues, but it confers a strong hydrophilicity, which can increase the solubility of drugs and improve the stability of ACPs [93]. Therefore, PEG modification can also be regarded as a new method of modification; this method merits further research.

#### 4.2.4. Glycosylation modification

Glycosylation of polypeptides is a process of linking sugars to specific amino acids by glycosyltransferases to form glycosidic bonds [94]. Glycosylation and phosphorylation are both post-translational modifications of proteins and involve many biological processes. Glycosylation increases the diversity of proteins and/or peptides and expands their functional range [95]. Glycosylation of short peptides does not necessarily lead to the production of effective ACPs, sometimes leading to loss of activity or function [96]. Studies have shown that five glycopeptides, viz., 8a–8e, were formed by combining monosaccharides with R-lycosin-I. The IC_50_ value of 8a was 37.4 µM for normal HEK-293T cells and 9.6 µM for lung cancer cells A549, which was lower than that of the template peptide [70]. In addition, some studies indicate that Aurein 1.2 peptide, IIb peptide and BMAP-28m peptide have selective cytotoxicity against MX-1 and MCF-7 breast cancer cells. After modification of these three ACPs by O-glycosylation, the Buforin IIb peptide obtained significantly inhibited the growth of both breast cancer cells, with IC_50_ values lower than 8 µM and low cytotoxicity to normal cells at this IC_50_. However, the inhibitory effect of the other two modified peptides on the two types of tumour cells was slightly worse, with IC_50_ values all being greater than 16 µM [97]. Glycosylation modification can be tried as a method of modification of ACPs, but as the effects vary from case to case, the results may be unpredictable or surprising.

#### 4.2.5. Palmitoylation modification

Palmitoylation is a process in which the 16-carbon saturated fatty acid, palmitate, forms a stable fatty acid amide chain through a reversible thioester chain [98]. Protein S-acylation involves combination of fatty acids with the protein for a reversible process of enzymatic modification after translation, and is driven by a series of protein acyltransferases; by adjusting the position of the protein, S-acylation plays an important role in the transport and function of the palmitoylation modified main protein [99,100]. C16-KKK-NH2 and C16-P_Cat_P_Hex_P_Hex_P_Cat_-NH2, which are an aliphatic tripeptide and aliphatic tetrapeptide, respectively, were obtained by S-palmitoylation of C14-NH4; the IC_50_ values of the former for human breast cancer cells MDA-MB-231 and JIMT-1, pancreatic cancer cells MiaPaCa2, and prostate cancer cells DU145 were 30.0 µM, 10.0 µM, 9.0 µM and 16.0 µM, respectively; for the latter the IC_50_ values were 12.5 µM, 17.0 µM, 16.5 µM and 23.0 µM, respectively [71]. When compared with before modification values, they are slightly lower, but the effect was limited. However, on performing a human erythrocyte haemolysis test, the two ACPs still showed weak haemolysis only when the concentration was 100.0 µM. R-C_12_, R-C_14_, R-C_16_, R-C_18_ and R-C_20_, which are five lipopeptides, were obtained by introducing 12–20 carbon fatty acids into R-lycosin-I for palmitoylation; these had suppressive effects on the A549 lung cancer cell. The best effect was seen with R-C_16_, with IC_50_ value of 5.0 µM. Compared with the IC_50_ value of 20.0 µM of R-lycosin-I, its anti-cancer effect obviously improved, but at the same time, at this IC_50_, both R-C_16_ and R-lycosin-I caused high toxicity to normal human cells [101]. It can be seen that if palmitoylation of ACPs can effectively reduce their cytotoxicity, their anti-tumour activity is not significantly improved. If this modification can significantly improve the anti-tumour activity of ACPs, it may not be effective in overcoming cytotoxicity.

## 5. Future prospects

The continuous augmentation and enrichment of ACP-related research is a strong positive signal in the research and development of new anti-tumour drugs; however, due to the special anti-tumour mechanisms of ACPs, their activity, toxicity and targeted efficacy need further improvement. With the development of modern medicine, science and technology, the reconstruction and modification of ACPs have also achieved gratifying results. Nonetheless, every single method still has its own limitations. Therefore, the evaluation strategy of ACPs should be sufficiently comprehensive to attain maximum efficiency. Constant exploration and finding solutions to the many negative effects are warranted so as to create a new screening system of ACPs. Collectively, additional research is needed to better guide further modification and application development of specific ACPs.

## Data accessibility

This article does not contain any additional data.
